# Nurses’ roles and responsibilities in suicide prevention: a scoping review

**DOI:** 10.1186/s12912-025-04009-5

**Published:** 2025-10-22

**Authors:** Annette Riedel, Stephanie Feinauer, Erik Jacob, Pia Madeleine Haug, Karen Klotz, Thomas Heidenreich

**Affiliations:** Faculty of Social Work, Education and Nursing Sciences, Esslingen University of Applied Sciences, Flandernstraße 101, 73732 Esslingen, Germany

**Keywords:** Nurse, Suicide prevention, Suicide, Role, Responsibility

## Abstract

**Background:**

Globally, suicide remains a major public health concern. As frontline caregivers, nurses play a crucial role in identifying and supporting those at risk. Despite their central role, the specific contribution of nurses to suicide prevention across the suicide prevention continuum and various care settings has not been mapped comprehensively. This scoping review aims to identify potential roles and responsibilities of nurses in suicide prevention within adult care settings from an international perspective.

**Methods:**

The review followed the methodological framework for scoping reviews outlined by the Joanna Briggs Institute. A comprehensive search of peer-reviewed empirical studies, case reports, guidelines, standards, policy papers, discussion papers and professional codes was conducted in MEDLINE, CINAHL and PsycInfo.

**Results:**

A total of *n* = 25 sources published between 1993 and 2024 in countries across Europe, Asia, North America and Australia were included. Six overarching nursing roles were identified: *Early Detection and Risk Assessment*, *Relationship-Building and Communication*, *Education and Advocacy*, *Risk Management*, *Interdisciplinary Collaboration* and *Personal and Professional Self-Management*. Each role encompasses specific responsibilities, reflecting the complex and multifaceted nature of nurses’ involvement. The findings further indicate that nurses contribute to universal, selective and indicated suicide prevention strategies.

**Conclusions:**

Nurses play a crucial role in suicide prevention. However, to fully realize their potential, institutional and systemic changes are required, including enhanced training, stronger organizational support and formal recognition at the policy-level. This serves the best interests of individuals at risk for suicide by holistically addressing their care-related support needs. Future research should focus on evaluating the implementation and effectiveness of nurses’ contribution to suicide prevention across diverse cultural and healthcare contexts, with the aim of strengthening nurses’ role in suicide prevention globally.

**Supplementary Information:**

The online version contains supplementary material available at 10.1186/s12912-025-04009-5.

## Background

Suicide remains a significant and urgent public health issue, claiming the lives of over 720,000 people across the globe each year [1]. *Suicidality* is understood as a multifaceted phenomenon that encompasses “a range of behaviours that include thinking about suicide (or ideation), planning for suicide, attempting suicide and suicide itself” [2, p. 12]. In the context of nursing care, the wish to die is a relatively common phenomenon among care recipients, especially among older adults [3–5]. This desire is recognized as an expression of personal suffering that can range from a passive wish to allow death to occur naturally, to an active wish to hasten death when the suicidal urge increases. Internationally, the wish to hasten death has received growing attention alongside the expanding legalization of euthanasia and assisted suicide [6–8]. As such, euthanasia refers to “a physician (or other person) intentionally killing a person by the administration of drugs, at that person’s voluntary and competent request.” [9, p. 108] In contrast, assisted suicide is distinguished by the fact that the “authority of action lies with the person who wants to terminate his or her life.” [9, p. 108–109] For the purposes of this scoping review, these practices are collectively referred to as Medical Assistance in Dying (MAiD), reflecting the complex and varied legal frameworks worldwide. Importantly, the desire to die is not commonly classified as suicidality in the traditional psychiatric sense, because it may differ from suicide from medical, legal, and ethical perspectives [10–12]. Nevertheless, it remains relevant to suicidality, as it can serve as an early warning sign for suicide risk and may require preventive interventions [13, 14]. This scoping review therefore explores phenomena such as the desire to die, MAiD and suicidality, as defined above, across a variety of adult care settings. Here, ‘adult care settings’ refers to all care contexts involving individuals aged 18 years and older, including but not limited to psychiatric care, with particular attention to high-risk groups such as older adults.

There is broad consensus that suicide can be prevented through targeted and evidence-based intervention strategies [15–18]. *Suicide prevention* is recognized as a shared societal responsibility that requires coordinated, multidisciplinary collaboration and the active involvement of various stakeholders at community and population levels [2, 19]. In this context, the WHO [2, 19] recommends a multi-level prevention approach, which includes universal measures addressing the general population, selective interventions for identified high-risk groups, and indicated measures targeting individuals at acute risk for suicide. Suicide prevention is of particular relevance for individuals with severe and chronic mental or physical illnesses as well as those who receive long-term care [20–27]. In particular, older adults in need of care constitute a high-risk population and should be specifically targeted by tailored suicide prevention strategies [28–30].

Nurses are widely recognized as frontline gatekeepers in the identification and management of individuals at risk for suicide [31–34]. Their close, ongoing contact with care recipients and the trusted relationships they build often lead nurses to encounter individuals expressing a desire to die [35, 36], or requesting MAiD [37–40]. Additionally, research indicates that nurses also face suicidality in a traditional sense across a variety of care settings [41–44].

For the purpose of this scoping review, ‘a *nurse*’ is defined in accordance with the International Council of Nurses (ICN) [45] as “a professional who is educated in the scientific knowledge, skills and philosophy of nursing, and regulated to practice nursing based on established standards of practice and ethical codes” [45, p. 45]. The ICN [45, 46] further emphasizes the essential roles and responsibilities of nurses, highlighting their vital contributions to health promotion, illness prevention, the alleviation of suffering, and the enhancement of health literacy. Through evidence-based practice, the development of trusted relationships, informed decision-making, and risk management, nurses play a crucial role in safeguarding individuals in need of care [45].

While some reviews^1^ examine suicide care and interventions from a nursing perspective [32, 47–50], none provide a comprehensive exploration of the potential roles and responsibilities of nurses in suicide prevention. To address this gap, this scoping review was conducted with the aim of identifying nurses’ potential roles and responsibilities in suicide prevention across adult care settings within an international context.

Guided by the research elements Population/Participants, Concept and Context (PCC) as outlined by Peters et al. [51], the following research question was developed and explored:

What are nurses’ roles and responsibilities in suicide prevention across adult care settings within an international context?

## Methods

### Design

A scoping review approach was selected to identify potential roles and responsibilities of nurses in suicide prevention [52]. Using a scoping review approach rather than a systematic review reflects the exploratory nature and conceptual ambiguity of the field. This scoping review is based on the framework outlined by Peters et al. [51] and includes the following steps: (1) Defining the objective and research question, (2) Establishing criteria for selecting relevant sources, (3) Outlining the planned approach to searching, selecting, extracting and presenting evidence, (4) Identifying relevant sources, (5) Applying inclusion/exclusion criteria to choose eligible sources, (6) Collecting relevant data from included sources, (7) Analyzing extracted data in line with the research objective, (8) Organizing and reporting the findings and (9) Summarizing findings, drawing conclusions and discussing implications. An a priori protocol was developed and registered in October 2024 on Open Science Framework (10.17605/OSF.IO/QYXSR). This scoping review was reported in accordance with the PRISMA Extension for Scoping Reviews (PRISMA-ScR), as outlined by Tricco et al. [53].

### Eligibility criteria

The inclusion and exclusion criteria were developed during the pre-registration phase, considering both content-related and formal methodological aspects. Following the recommendations by Peters et al. [51], the content-related inclusion criteria were defined in alignment with the core elements of the research question and aim. Accordingly, the scoping review includes evidence sources that meet the following content-related criteria:

- **Population/Participants**: Evidence sources were included if they reflected the perspectives of nurses with varying levels of professional qualification or if nurses were actively involved as participants. Sources were excluded if the population included other healthcare professionals alongside nurses and the data specific to nurses could not be extracted and analyzed separately.
- **Concept**: Evidence sources were included if they focused on the roles and responsibilities of nurses in suicide prevention, in the context of suicidality, desire to die and MAiD. Sources were excluded if they addressed suicidality among nurses themselves, the training or educational needs of nurses related to specific interventions or programs, or the testing or evaluation of particular interventions, programs, or instruments.
- **Context**: Evidence sources were included if they focused on adult care settings in an international context. By adult care settings, we refer to any care context involving individuals aged ≥ 18 years. All adult care settings where suicide, the desire to die, or MAiD may be relevant were considered, including but not limited to psychiatric care. Sources were excluded if they examined non-care settings or focused exclusively on suicide prevention among children or adolescents. The latter were excluded because both suicidal behaviour [54] and preventive interventions [55] may differ in these groups when compared to adults and because MAiD is legally restricted to individuals aged ≥ 18 years in most countries [7].

The formal methodological inclusion and exclusion criteria include “types of evidence sources”, “language” and “publication date”. The scoping review includes evidence sources that meet the following formal-methodological criteria:

- **Types of Evidence Sources**: As suggested by Peters et al. [56], a variety of study designs were considered for inclusion to ensure a comprehensive review of the existing scientific literature on the topic. In line with the research question, empirical studies – including qualitative, quantitative, and mixed-method research, case reports – published in peer-reviewed journals as well as reviews incorporating peer-reviewed empirical studies were considered relevant for inclusion. In addition, guidelines, standards, policy papers, discussion papers, and professional codes were also deemed appropriate for inclusion.
- **Language**: Evidence sources published in English and German were included, based on the language proficiency of the research team.
- **Publication date**: Evidence sources published in any year were considered for inclusion. Sources were eligible for inclusion up to November 22nd 2024.

### Search strategy

A comprehensive literature search was conducted in October and November 2024 by SF in the electronic databases MEDLINE via EBSCOhost, CINAHL via EBSCOhost, and PsycINFO via EBSCOhost to locate published and unpublished evidence sources [51]. The search strategy was developed in three stages [51]. As a first step, initial searches were conducted in MEDLINE via EBSCOhost and CINAHL via EBSCOhost by searching the terms “nurse“, “suicide“, “prevention“, “role“, “responsibility“ and “relationship“ as well as synonyms and related terms. Subsequent to the initial search, text words in the titles and abstracts of the retrieved publications were analyzed, along with the indexing terms assigned to them.

As a second step, the identified terms were used to construct three distinct search strings – one for each database – by applying the Boolean operators “AND“ and “OR“, together with database-specific keywords and search syntax. The search strings were pretested, evaluated and refined according to the PRESS 2015 Guideline Evidence-Based Checklist by McGowan et al. [57]. Additionally, relevant publications identified during the initial searches were incorporated to validate and further optimize the search strategy [51]. Following this, a comprehensive second search was conducted in each included database using these tailored search strings. The complete search strategies for all databases are presented in Supplementary File 1. As a third step, additional sources were identified by screening the reference lists of all studies included after full-text review.

### Screening and selection

Based on the predefined inclusion and exclusion criteria outlined above, the selection of papers was independently conducted by two reviewers (SF, EJ) in December 2024 and January 2025. This process was guided by the PRISMA-ScR statement [58] and comprised two stages: an initial screening of titles and abstracts, followed by full-text screening. Any disagreements between the reviewers were resolved by consensus, or, when consensus could not be reached, through consultation with the review team [51].

Prior to initiating the title and abstract screening, a total of *n* = 1.465 records were retrieved, using the developed search strings and imported into Rayyan [59]. Duplicates were identified automatically through Rayyan and manually removed by SF, resulting in *n* = 952 records eligible for title and abstract screening. In accordance with Peters et al. (2020) [51], pilot testing of the source selection process was conducted prior to initiating full source selection, with the aim of refining the selection guidance. The selection guidance was pilot tested using a random sample of *n* = 25 records, which were independently screened by two raters (SF, EJ) according to the predefined eligibility criteria and accompanying definitions. Subsequently, the raters met to resolve any discrepancies and revised the eligibility criteria and guidance as necessary. Full dataset screening commenced only after achieving a minimum inter-rater agreement of 75%. The finalized screening guidance is provided in Supplementary File 2. Relevant review articles were selected specifically to enable hand searching of their reference lists. However, they were excluded from data extraction and analysis. Supplementary File 3 presents a brief overview of the excluded sources, including the review articles.

To assess inter-rater reliability, Krippendorff’s Alpha was applied [60], using the Krippendorff’s Alpha Calculator developed by Marzi et al. [61]. After screening titles and abstracts, Krippendorff’s Alpha was calculated at 0.840, reflecting a satisfactory level of inter-rater agreement for inclusion decisions. After the full-text screening, the coefficient increased slightly to 0.866.

### Data extraction and analysis

The data extraction process was conducted in February and March 2025 using the software MAXQDA. Prior to the final extraction, an extraction table was developed during the preregistration phase and further refined during the review phase [51]. To ensure that all relevant data were captured, a pilot test was independently conducted by two raters (SF and EJ) on a random sample of *n* = 5 sources. Following the approach outlined by Peters et al. [51], the final data extraction was performed by SF and the extracted data were subsequently verified by the rater team. The finalized extraction guidance, provided in Supplementary File 4, includes general information about the sources (author/s, year of publication), their characteristics (country of origin, publication type, population/participants, context/setting), and the results relevant to the review question (concept).

In line with the aims and research questions of this review, the analysis was conducted in April and May 2025 using MAXQDA, with a focus on identifying potential roles and responsibilities of nurses in the context of suicide prevention. The analysis utilized the basic qualitative content analysis approach as described by Pollock et al. [62]. In line with the inductive methodology underlying this approach, the data were systematically coded and organized into specific categories emerging from the material. These categories emerged through open coding of the dataset, based directly on the text [53]. Findings from the analysis are reported narratively.

## Results

### Search results

**Fig. 1:**
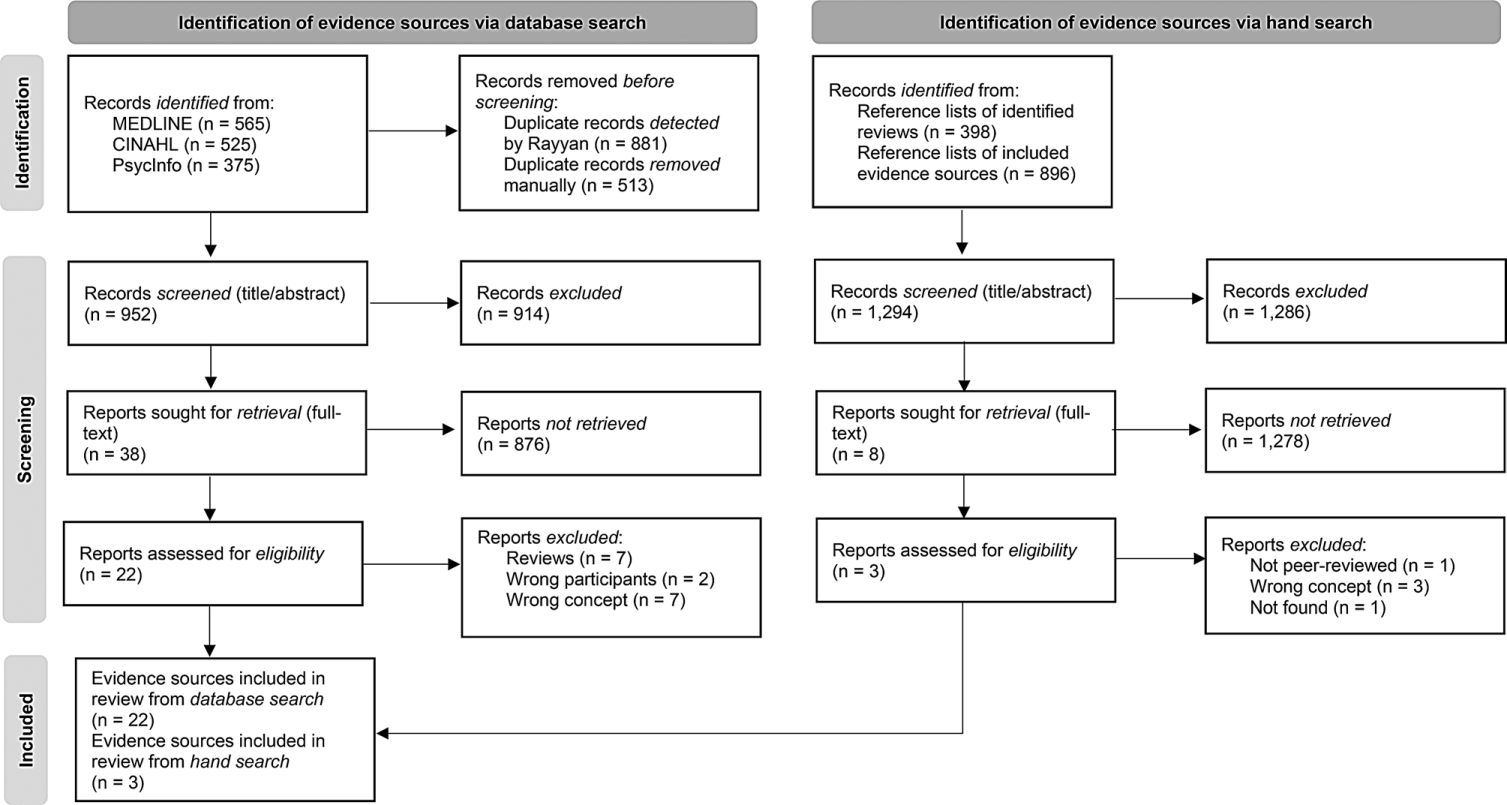
PRISMA flow diagram detailing the database search and study selection process. Adapted from: https://www.prisma-statement.org [Accessed March 17 2025], licensed under CC BY 4.0 (https://creativecommons.org/licenses/by/4.0/)

A comprehensive search across multiple databases yielded *n* = 1.465 records in total, including *n* = 565 from MEDLINE via EBSCO, *n* = 525 from CINAHL via EBSCO and *n* = 375 from PsycInfo via EBSCO. Figure 1 provides an overview of the entire identification and inclusion process. The screening tool Rayyan was employed to identify duplicate records, detecting *n* = 881 potential duplicates. After manual verification by SF, *n* = 513 duplicates were confirmed and removed. The deduplication process resulted in *n* = 952 sources of evidence remaining for title and abstract screening. Following the title and abstract screening, *n* = 914 records were excluded for not meeting the predefined inclusion criteria. A total of *n* = 38 sources were considered potentially relevant and proceeded to full-text screening. Of these, *n* = 16 were excluded as they failed to meet one or more inclusion criteria related to population/participants, context, or concept or did not match the predefined publication type. Figure 1 provides detailed reasons for exclusion. Ultimately, *n* = 22 sources of evidence retrieved from the database search met all inclusion criteria and were included in the scoping review. Additionally, the reference lists of these *n* = 22 sources and *n* = 7 identified relevant review articles were hand searched, leading to the inclusion of *n* = 3 further sources of evidence. In total, *n* = 25 sources of evidence were included in the scoping review.

### Characteristics of included sources of evidence

The included evidence was published between 1993 and 2024 in countries across Europe, North America, Asia and Australia. It comprises *n* = 1 discussion paper [63], *n* = 1 position paper [64], *n* = 14 qualitative studies [42, 43, 65–76], *n* = 4 quantitative studies [77–80], *n* = 1 mixed-methods study [81], *n* = 2 case studies [82, 83] and *n* = 2 guidelines [34, 84]. *N* = 1 qualitative study addressed suicide prevention in the context of MAiD through assisted suicide [74]. Another qualitative study specifically focused on nursing students [76]. Table 1 provides details of the included sources of evidence.

**Table 1:** Characteristics of included evidence sources

Reference	Country	Type of evidence source	Population/ participants	Context (Setting)	Concept (Extracted roles)
(Anderson and Jenkins 2006) [63]	England	Discussion Paper	Nursing Profession	Mental Health Nursing	Early Detection and Risk Assessment Education and Advocacy Risk Management Interdisciplinary Collaboration
(Chijiiwa and Ishimura 2024) [65]	Japan	Qualitative Study	General Home Visiting Nurses (GHVN)	General Home Visiting Nursing Facilities that care for Patients with Physical Illnesses	Early Detection and Risk Assessment Relationship-Building and Communication Interdisciplinary Collaboration
(Darnell et al. 2023) [66]	USA	Qualitative Study	*N* = 13 Acute and Intensive Care Nurses	Urban level 1 Trauma Center	Relationship-Building and Communication Risk Management Interdisciplinary Collaboration
(Doyle et al. 2007) [77]	Ireland	Quantitative Study	*N* = 42 Emergency Department Nurses	*N* = 2 Teaching Hospitals	Early Detection and Risk Assessment Relationship-Building and Communication Risk Management Interdisciplinary Collaboration
(Eriksson et al. 2024) [43]	Sweden	Qualitative Study	*N* = 10 Geriatric Nurses	*N* = 4 Municipalities in Geriatric Nursing	Early Detection and Risk Assessment Relationship-Building and Communication Education and Advocacy Risk Management Interdisciplinary Collaboration Personal and Professional Self-Management
(Hagen et al. 2017) [67]	Norway	Qualitative Study	*N* = 8 Mental Health Nurses	*N* = 5 Psychiatric Wards within *N* = 2 Hospitals	Early Detection and Risk Assessment Risk Management Personal and Professional Self-Management
(Jansson and Graneheim 2018) [42]	Sweden	Qualitative Study	*N* = 8 Registered Nurses, *N* = 4 Enrolled Nurses	Outpatient Unit in a Psychiatric Clinic	Early Detection and Risk Assessment Education and Advocacy Risk Management Personal and Professional Self-Management
(Kusheba and Mulvihill 2018) [82]	USA	Case Study	Clinical Nurse Leader	Hospice Home Care	Early Detection and Risk Assessment Relationship-Building and Communication Risk Management Interdisciplinary Collaboration Personal and Professional Self-Management
(Lees et al. 2014) [81]	Australia	Mixed-methods Study	Survey: *N* = 87 Mental Health Nurses Interviews: *N* = 11 Mental Health Nurses	Community and Inpatient Settings within a Public Mental Health Service	Early Detection and Risk Assessment Relationship-Building and Communication Risk Management
(Litta et al. 2024) [78]	Italy	Quantitative Study	*N* = 84 Nurses	Medical, Surgical, Critical and Emergency Services	Risk Management Interdisciplinary Collaboration
(Marutani et al. 2016) [68]	Japan	Qualitative Study	*N* = 17 Public Health Nurses	*N* = 14 Cities in Metropolitan Regions	Education and Advocacy Interdisciplinary Collaboration
(Öztürk and Hiçdurmaz 2023) [69]	Turkey	Qualitative Study	*N* = 33 Oncology Nurses	*N* = 3 State, *N* = 3 University and *N* = 2 Private Hospitals	Early Detection and Risk Assessment Risk Management
(Puntil et al. 2013) [64]	USA	Position Paper	Psychiatric Mental Health Nurse Generalists	Hospital, Inpatient Psychiatric Units	Risk Management Interdisciplinary Collaboration
(Registered Nurses’ Association of Ontario (RNAO) 2009) [34]	Canada	Best Practice Guideline	Nurses	Nursing Practice	Early Detection and Risk Assessment Relationship-Building and Communication Education and Advocacy Risk Management Interdisciplinary Collaboration Personal and Professional Self-Management
(Reid and Long 1993) [79]	Northern Ireland	Quantitative Study	*N* = 50 Psychiatric Nurses	Acute Wards in an Psychiatric Training Hospital	Early Detection and Risk Assessment Relationship-Building and Communication Education and Advocacy Risk Management Interdisciplinary Collaboration Personal and Professional Self-Management
(Serafica et al. 2023) [83]	USA	Case Study	Primary Care Providers	Nursing Home	Early Detection and Risk Assessment Risk Management Interdisciplinary Collaboration
(Sun et al. 2005) [70]	Taiwan	Qualitative Study	*N* = 5 Psychiatric Nurses	*N* = 3 Psychiatric Hospitals	Early Detection and Risk Assessment Relationship-Building and Communication Education and Advocacy Risk Management Interdisciplinary Collaboration
(Sun et al. 2006) [80]	Taiwan	Quantitative Study	*N* = 15 Nurses *N* = 15 Patients	*N* = 3 Hospitals, *N* = 3 Acute Psychiatric Wards and *N* = 1 Psychiatric Stress Ward	Early Detection and Risk Assessment Relationship-Building and Communication Risk Management
(Vandewalle et al. 2019) [71]	Belgium	Qualitative Study	*N* = 26 Psychiatric Nurses	*N* = 2 Wards in *N* = 4 Psychiatric Hospitals	Early Detection and Risk Assessment Relationship-Building and Communication Risk Management Personal and Professional Self-Management
(Vandewalle et al. 2019b) [72]	Belgium	Qualitative Study	*N* = 19 Nurses with Experience in caring for Patients with Suicidal Ideation	*N* = 4 Psychiatric Hospitals	Early Detection and Risk Assessment Relationship-Building and Communication Education and Advocacy Risk Management Personal and Professional Self-Management
(Vandewalle et al. 2020) [73]	Belgium	Qualitative Study	*N* = 28 Psychiatric Nurses	*N* = 13 Adult Wards in *N* = 4 Psychiatric Hospitals	Early Detection and Risk Assessment Relationship-Building and Communication Education and Advocacy Risk Management Interdisciplinary Collaboration Personal and Professional Self-Management
(Volker 2003) [74]	USA	Qualitative Study	*N* = 24 Oncology Nurses	Care of Terminally Ill Patients	Education and Advocacy Risk Management
(Wärdig et al. 2022) [75]	Sweden	Qualitative Study	*N* = 15 Registered Nurses that work in Primary Health Care for minimum 1 year	Primary Health Care	Early Detection and Risk Assessment Interdisciplinary Collaboration
(Zaleski et al. 2018) [84]	USA	Clinical Practice Guideline	Nurses	Emergency Departments	Early Detection and Risk Assessment
(Zohn 2022) [76]	USA	Qualitative Study	*N* = 14 Nursing Students	*N* = 2 Universities, Psychiatric and Mental Health Care	Early Detection and Risk Assessment Relationship-Building and Communication Education and Advocacy Risk Management Personal and Professional Self-Management

### Potential roles and responsibilities of nurses in suicide prevention

The findings from the analyzed sources allow for a clearer specification of nurses’ roles and responsibilities in the context of suicide prevention. Table 2 presents six themes that represent potential roles of nurses in suicide prevention. These roles include: 3.3.1 *Early Detection and Risk Assessment*, 3.3.2 *Relationship-Building and Communication*, 3.3.3 *Education and Advocacy*, 3.3.4 *Risk Management*, 3.3.5 *Interdisciplinary Collaboration*, and 3.3.6 *Personal and Professional Self-Management*. The table also indicates which types of evidence contributed to each theme and specifies the responsibilities nurses may be expected to assume within these roles. A narrative summary of the analysis is provided in the following section.

**Table 2:** Overview of identified nursing roles and responsibilities in suicide prevention

Potential roles in suicide prevention	Potential role-specific responsibilities in suicide prevention	Contribution to themes by source type
3.3.1 Early Detection and Risk Assessment	• Recognize increased vulnerability • Screen individual risk factors and warning signs • Conduct an in-depth assessment	• Case Study: *n* = 2 [82, 83] • Discussion Paper: *n* = 1 [63] • Guidelines: *n* = 2 [34, 84] • Mixed-Methods Study: *n* = 1 [81] • Position Paper: *n* = 0 • Qualitative Study: *n* = 11 [42, 43, 65, 67, 69–73, 75, 76] • Quantitative Study: *n* = 3 [77, 79, 80]
3.3.2 Relationship-Building and Communication	• Cultivate a relationship-enhancing attitude • Develop and maintain relationships • Foster open communication	• Case Study: *n* = 1 [82] • Discussion Paper: *n* = 0 • Guidelines: *n* = 1 [34] • Mixed-Methods Study: *n* = 1 [81] • Position Paper: *n* = 0 • Qualitative Study: *n* = 8 [43, 65, 66, 70–73, 76] • Quantitative Study: *n* = 3 [77, 79, 80]
3.3.3 Education and Advocacy	• Provide information and counseling to care recipients and their relatives • Conduct research and implement public health strategies for suicide prevention • Raise awareness	• Case Study: *n* = 0 • Discussion Paper: *n* = 1 [63] • Guidelines: *n* = 1 [34] • Mixed-Methods Study: *n* = 0 • Position Paper: *n* = 0 • Qualitative Study: *n* = 8 [42, 43, 68, 70, 72–74, 76] • Quantitative Study: *n* = 1 [79]
3.3.4 Risk Management	• Minimize risk factors and enhance protective factors • Alleviate suffering • Create a safe environment	• Case Study: *n* = 2 [82, 83] • Discussion Paper: *n* = 1 [63] • Guidelines: *n* = 1 [34] • Mixed-Methods Study: *n* = 1 [81] • Position Paper: *n* = 1 [64] • Qualitative Study: *n* = 11 [42, 43, 66, 67, 69–74, 76] • Quantitative Study: *n* = 4 [77–80]
3.3.5 Interdisciplinary Collaboration	• Exchange information • Support team members • Coordinate with relevant experts and help centers	• Case Study: *n* = 2 [82, 83] • Discussion Paper: *n* = 1 [63] • Guidelines: *n* = 1 [34] • Mixed-Methods Study: *n* = 0 • Position Paper: *n* = 1 [64] • Qualitative Study: *n* = 8 [43, 65, 66, 68, 70, 73, 75, 76] • Quantitative Study: *n* = 3 [77–79]
3.3.6 Personal and Professional Self-Management	• Navigate value-based conflicts • Cope with challenges, fears and uncertainties • Practice self-awareness, reflection and emotional debriefing	• Case Study: *n* = 1 [82] • Discussion Paper: *n* = 0 • Guidelines: *n* = 1 [34] • Mixed-Methods Study: *n* = 0 • Position Paper: *n* = 0 • Qualitative Study: *n* = 6 [42, 43, 67, 71–73] • Quantitative Study: *n* = 1 [79]

#### Early detection and risk assessment

*n* = 20 of the reviewed sources highlight the pivotal role of nurses in suicide prevention regarding the early identification, screening and assessment of suicide risk [34, 42, 43, 63, 65, 67, 69–73, 75–77, 79–84]. This theme is supported by a broad range of evidence sources, including two guidelines [34, 84], one discussion paper [63], two case studies [82, 83], one mixed-methods study [81], three quantitative studies [77, 79, 80], and eleven qualitative studies [42, 43, 65, 67, 69–73, 75, 76].

In this context, nurses are expected to recognize especially vulnerable population groups and identify risk factors [43, 63]. Furthermore, nurses report about their responsibility in detecting warning signs associated with suicidality such as behavior changes or desire to die statements [42, 43, 65, 67, 70–73, 80]. In addition, to identifying an individual’s suicide risk, nurses take responsibility for conducting a more in-depth assessment, which includes reaching a shared understanding of the care recipient’s risk by exploring factors such as severity, underlying motives, personal meanings, causes, the persistence and seriousness of suicidal thoughts, intent to self-harm, and existing protective factors [42, 43, 63, 65, 71–73, 75, 80]. Some evidence suggests that nurses play a less prominent role compared to physicians and that their responsibility in suicide identification, screening and assessment is not universally recognized as a standard part of nursing practice [69, 75].

#### Relationship-building and communication

*n* = 14 of the reviewed sources emphasize the vital role of nurses in establishing relationships and communication with care recipients at risk of suicide in the context of suicide prevention. [34, 43, 65, 66, 70–73, 76, 77, 79–82]. This theme is supported by a wide range of evidence sources, including one guideline [34], one case study [82], one mixed-methods study [81], three quantitative studies [77, 79, 80], and eight qualitative studies [43, 65, 66, 70–73, 76].

Evidence indicates that a key responsibility of nurses is to cultivate a relationship-enhancing attitude, which is characterized by qualities such as understanding, open-mindedness, responsiveness, interest, acceptance, empathy, collaboration, authenticity, transparency, presence and respect for the individual’s dignity [43, 70–73, 77, 81, 82]. Furthermore, evidence indicates that nurses contribute to suicide prevention through the relationships they develop and maintain with care recipients – relationships often described as close, trusting, collaborative, and therapeutic [43, 65, 66, 70, 73, 80]. These relationships can be preventive in nature, enhance the effectiveness of suicide prevention efforts, facilitate the expression of thoughts and emotions, promote positive interactions, instill a sense of hope for the future, and provide safety for the individual in need of care [71–73, 80, 81]. Facilitating open communication about suicidal ideation is also identified as a central responsibility of nurses within the scope of suicide prevention [66, 70–73, 81, 82].

#### Education and advocacy

As highlighted by *n* = 11 sources, nurses are also shown to play an important role in both educational efforts and advocacy in the context of suicide prevention [34, 42, 43, 63, 68, 70, 72–74, 76, 79]. This theme is supported by a moderate range of evidence sources, including one guideline [34], one discussion paper [63], one quantitative study [79], and eight qualitative studies [42, 43, 68, 70, 72–74, 76].

In this role, evidence indicates that nurses are responsible for offering information and guidance to individuals affected by suicidality, a desire to die, or MAiD, as well as to their relatives and the broader public [43, 63, 70, 74, 76]. This includes, for example, teaching journalists to promote responsibility in media reporting [63], counselling and emotional support [43, 70]. Furthermore, the evidence shows that nurses are involved in conducting research on suicide and implementing public health strategies for suicide prevention [68]. Finally, the reviewed evidence suggests that nurses consider it part of their professional responsibility to raise awareness of suicide risk among individuals in need of care and within the interdisciplinary team, as well as to address the needs of those affected [68, 70, 72–74, 76].

#### Risk management

Another significant role of nurses in suicide prevention, as identified in *n* = 21 sources, is providing risk management, which encompasses preventive interventions and the containment of potential suicide risks [34, 42, 43, 63, 64, 66, 67, 69–74, 76–83]. This theme is supported by a broad range of evidence sources, including one guideline [34], one discussion paper [63], one position paper [64], two case studies [82, 83], one mixed-methods study [81], four quantitative studies [77–80], and eleven qualitative studies [42, 43, 66, 67, 69, 71–74, 76].

A central responsibility emerging from the evidence within this role is the minimization of risk factors and the strengthening of protective factors [43, 66, 67, 70–74]. Risk management may include promoting a sense of meaning, joy and positivity in life [43, 72, 73], promoting social connection [43, 72, 73], fostering coping strategies such as physical activity [72], and support care recipients in dealing with difficult situations [73]. Furthermore, the evidence indicates that nurses have a responsibility in risk management by alleviating suffering and addressing the diverse needs of individuals in need of care [63, 64, 67–70, 72–74, 80–82]. This responsibility may include:

- *Management of psychological symptoms*, for example through engaging in therapeutic interventions to manage mental disorders and instilling hope [63, 67, 70, 72];
- *Management of physical symptoms*, for example through pain management [70, 74];
- *Management of social symptoms*, for example through fostering social connectedness and encouraging support from professionals, family and community networks [63, 68];
- *Management of spiritual symptoms*, for example through involving spiritual care experts [69, 82].

Moreover, the creation of a safe environment for individuals at risk of suicide is identified as a part of nurses’ professional responsibility [42, 43, 66, 67, 70, 72, 73, 76, 77, 80, 82]. Fulfilling this responsibility may require generating safety plans and making agreements with individuals [42, 66, 70, 72, 73], restricting access to means of suicide [43, 66, 70, 80, 82], conducting observations [43, 70, 72, 77, 81], and the usage of restrictive measures [70].

#### Interdisciplinary collaboration

*n* = 16 reviewed sources also highlight the important role of nurses in suicide prevention through collaboration with professionals from other disciplines and organizations [34, 43, 63–66, 68, 70, 73, 75, 77–79, 82, 83]. This theme is supported by a broad range of evidence sources, including one guideline [34], one discussion paper [63], one position paper [64], two case studies [82, 83], three quantitative studies [77–79], and eight qualitative studies [43, 65, 66, 68, 70, 73, 75, 76].

Evidence suggests that, within interdisciplinary collaboration, nurses are responsible for the exchange of information with professional team members and relatives to support early identification of suicide risk, a thorough risk assessment and the development of targeted prevention strategies [43, 65, 70, 82]. Moreover, the evidence indicates that nurses hold a collaborative responsibility within their teams by providing mutual support, consulting with colleagues, and distributing responsibilities when working with individuals at risk of suicide [73, 75, 82, 83]. In addition, the reviewed sources of evidence emphasize the responsibility of nurses to direct individuals at risk for suicide or in crisis to relevant professionals and support centers [63, 66, 75, 77, 83].

#### Personal and professional self-management

Another pivotal role of nurses in suicide prevention identified in *n* = 9 sources involves practicing self-management both personally and professionally [34, 42, 43, 67, 71–73, 76, 79, 82]. This theme is supported by a moderate range of evidence sources, including one guideline [34], one case study [82], one quantitative study [79], and six qualitative studies [42, 43, 67, 71–73].

The reviewed evidence indicates that nurses are confronted with various value-based conflicts in the context of suicide prevention, which they must navigate as part of their professional responsibility [42, 72, 73, 82]. Moreover, nurses report facing a range of challenges in the context of caring for individuals at risk for suicide, including being confronted with intense emotions and disclosures of suicidal ideation, fears of being held personally responsible in the event of suicide, feelings of isolation in bearing responsibility, uncertainty regarding roles and appropriate procedures [42, 43, 67, 72, 76, 82]. In this context, evidence suggests that nurses often feel that it is their responsibility to manage these challenges, fears and uncertainties in order to remain capable of taking appropriate action [42, 67, 71–73, 76]. Therefore, the evidence highlights the importance of nurses being aware of their own emotions and managing them appropriately, engaging in reflective practice and emotional debriefing, and upholding professional boundaries [67, 71–73].

#### Nurses’ roles and responsibilities across the suicide prevention continuum

**Fig. 2:**
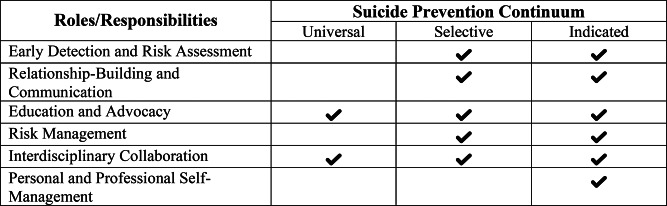
Nurses’ roles and responsibilities across the suicide prevention continuum

The findings of this scoping review illustrate that nurses hold a pivotal role across all three levels of suicide prevention proposed by the WHO [2, 19]: universal, selective and indicated This broad scope of engagement underscores the essential and multifaceted role and responsibility of nurses in comprehensive suicide prevention efforts internationally. Figure 2 illustrates how the identified roles align with each of these levels. In the context of universal suicide prevention, nurses contribute by raising awareness, promoting mental health, and reducing stigma, thereby strengthening protective factors within the broader population [68, 70–74, 76]. This is supported in particular by educational measures, advocacy and interdisciplinary cooperation. At the selective level, they are actively involved in the early identification of individuals at risk, recognizing warning signs, and initiating interventions to reduce risk [42, 43, 63, 65, 67, 70–73, 80]. Appropriate educational measures, advocacy and interdisciplinary cooperation also play an important role at this level. In indicated prevention, nurses support individuals affected by suicidality, desire to die or MAiD fostering trusted and therapeutic relationships, and participating in coordinated risk management [42, 43, 65–67, 70, 73, 80–82] The relevant skills and the necessary attitude are acquired and passed on through participation in training measures. The role and responsibility in the interdisciplinary team are also fundamental here. At this level, personal and professional self-management plays a significant role, as was made clear earlier.

## Discussion

### Summary and interpretation of findings

This scoping review aimed at identifying potential roles and responsibilities of nurses in suicide prevention within adult care settings from an international perspective. It includes *n* = 25 evidence sources, published between 1993 and 2024, originating from countries across Europe, North America, Asia and Australia, and covering a variety of healthcare contexts.

The findings of this scoping review illustrate that nurses hold a pivotal role across all three levels of suicide prevention proposed by the WHO [2, 19]. This broad scope of engagement underscores the essential and multifaceted role and responsibility of nurses in comprehensive suicide prevention efforts internationally. Importantly, although nurses act as gatekeepers in multi-level suicide prevention worldwide, their practice is inevitably influenced by country-specific legal, cultural, and professional frameworks. For instance, this is particularly relevant in the context of suicide preventive care in MAiD: while MAiD is increasingly legalized in many countries, it remains ethically contentious [7, 8], creating ethical challenges for nurses who also bear responsibility for suicide prevention [85]. Moreover, suicidality may be influenced by cultural factors, highlighting the need for nursing interventions that consider culture-specific risk and protective factors as well as warning signs for suicide [86, 87]. Professional qualifications and nurse competencies can vary significantly within and across countries (e.g., vocational training, higher education training) [88]. Given the nuanced nursing roles we identified in suicide prevention, responsibilities can and should therefore be adapted to diverse legal and ethical contexts as well as to differing levels of professional qualification.

Against this backdrop, the comprehensive synthesis of the multifaceted roles and responsibilities of nurses in suicide prevention within adult care settings in our scoping review highlights the depth of their involvement across diverse healthcare systems internationally. The identification of six key roles – 3.3.1 *Early Detection and Risk Assessment*, 3.3.2 *Relationship-Building and Communication*, 3.3.3 *Education and Advocacy*, 3.3.4 *Risk Management*, 3.3.5 *Interdisciplinary Collaboration*, and 3.3.6 *Personal and Professional Self-Management* – underscores the complexity of nursing contributions to suicide prevention and the essential nature of their work in this area. These roles are not only conceptually distinct but also vary in the strength of support by evidence type. Although most themes were supported by robust sources, including a best practice guideline and multiple qualitative and quantitative studies (Table 2), theme 3*.3.6*,* Personal and Professional Self-Management*, was supported by comparatively fewer empirical studies, underscoring the need for further research in this area.

The evidence consistently emphasizes the critical frontline position nurses hold in identifying suicide risk. Through regular and often prolonged patient contact, nurses are uniquely placed to observe subtle behavior and emotional changes, making *Early Detection and Risk Assessment* a core component of their practice. This finding is also supported by the results of previous reviews [32, 48]. However, despite this strategic positioning, some studies indicate a lack of standardized assessment tools and variable confidence levels among nurses in recognizing and evaluating suicidal ideation [77, 82]. This highlights a pressing need for enhanced training and consistent protocols to ensure nurses can respond effectively and confidently.

*Relationship-Building and Communication* emerged as another cornerstone role, with studies emphasizing the therapeutic impact of a trusting nurse-patient relationship [70–72, 80]. Open, non-judgmental communication is often the first step toward uncovering suicidal thoughts, yet this requires time, emotional investment, and organizational support—resources that may be limited in high-demand care environments. Therefore, institutional recognition and prioritization of relationship-based care in suicide prevention strategies are essential [47].

The role of *Education and Advocacy* reflects the broader societal responsibility nurses carry in mitigating stigma, educating individuals and relatives, and engaging in public health initiatives. While some studies document nurses taking proactive roles in these areas [68, 74, 76], others reveal gaps in knowledge and insufficient integration of suicide prevention into nurse education curricula. Given the increasing mental health burden globally [89], strengthening this role could amplify the preventative reach of nursing beyond clinical settings.

*Risk Management* responsibilities also illustrate the balancing act nurses perform between alleviating immediate distress associated with suicidality, desire to die and MAiD and maintaining a safe, therapeutic environment. Strategies range from ensuring environmental safety to strengthening protective factors like hope and social support [42, 43, 66, 67, 72, 73]. The diversity of approaches across countries and care settings, together with the influence of culture on suicidality, underscores the need for context-specific, culturally sensitive, and person-centered interventions to reduce suicide risk [86, 87]. Furthermore, interventions should also aim to support nurses in critically reflecting on their own biases toward racial, ethnic, and sexual minority care recipients, employing humility-informed frameworks [90].

*Interdisciplinary Collaboration* is widely recognized as vital for comprehensive suicide prevention. Nurses often act as coordinators and communicators, linking care recipients with appropriate services and professionals [43, 65, 66, 77, 82]. However, the effectiveness of this role is contingent upon organizational structures that support seamless information-sharing and integrated care pathways. Barriers such as poor communication channels, unclear role boundaries, and hierarchical team dynamics can limit the potential of this collaborative function.

Finally, *Personal and Professional Self-Management* points to the emotional impact and moral challenges nurses face in this area of care. The evidence illustrates that suicide prevention often involves exposure to distressing situations, ethical dilemmas, and emotional fatigue [43, 67]. Research shows that nurses may experience moral distress in the context of MAiD [85]. This may be particularly true in situations where nurses perceive that suicide preventive measures align more with the core values of nursing than MAiD, yet individuals in need of care nonetheless choose MAiD. Thus, to address ethical issues with regard to suicide prevention, institutions should foster an open ethical climate [85], where nurses can voice varying ethical viewpoints on the topics of suicidality, desire to die, MAiD and suicide prevention. Moreover, the emphasis on reflection, emotional debriefing, and value-based practice signals the importance of institutional mechanisms – such as adequate staffing and supervision – to support nurses’ mental health and resilience [47]. Failing to address these needs may lead to burnout, reduced quality of care, and attrition from the workforce.

### Strengths and limitations

One of the key strengths of this scoping review lies in its comprehensive and systematic approach to mapping the roles and responsibilities of nurses in suicide prevention across a wide range of international contexts. By including a broad variety of evidence sources this review captures the complexity and diversity of nursing practice in this field. The inclusion of studies published over three decades (1993–2024) further enhances the depth of analysis. Additionally, the review’s structured categorization of six core roles with clearly defined responsibilities offers a framework that can inform further research.

Despite these strengths, several limitations must be acknowledged. First, while the review sought international perspectives, the distribution of sources was uneven across regions, with some geographical areas underrepresented; potentially limiting the global generalizability of the findings. Second, our analysis focused on the types of evidence sources underpinning the identified themes rather than on a systematic critical appraisal of their quality. This is consistent with the methodological framework of scoping reviews [62] and appropriate given the limited evidence base in this field. Nonetheless, future systematic reviews are warranted to evaluate the quality of the available evidence. Third, the heterogeneity of the included evidence sources – varying in design, context, population, and terminology – posed challenges in synthesizing the data and drawing comparisons. Moreover, the scope was limited to adult care settings, which may exclude relevant insights from pediatric or adolescent populations where suicide prevention is also critical. Finally, the review may be subject to publication bias, as gray literature and non-English sources were not systematically included, potentially omitting valuable perspectives.

Overall, while this scoping review provides a valuable and timely overview of nurses’ roles and responsibilities in suicide prevention, its findings should be interpreted in light of these methodological constraints. Further research focused on region-specific suicide prevention care is needed to build on these insights and fill existing gaps.

### Implications for research and clinical practice

Overall, the roles identified in this review demonstrate that suicide prevention is not a singular intervention but a continuum of care that nurses are deeply embedded in, both clinically and ethically. The findings also suggest a need for organizational and societal changes to better equip and support nurses in these roles, including comprehensive training, organizational backing, and policy-level recognition of their central role in suicide prevention. Future research should continue to explore how these roles are implemented in various cultural and healthcare contexts, how nurses experience suicide prevention emotionally and ethically, and how best practices can be adapted and shared globally to strengthen the nursing contribution to suicide prevention.

In practice, there is a pressing need to integrate suicide prevention competencies into nursing curricula for initial, continuing and further training, ensuring that nurses enter the workforce with the skills and confidence to engage effectively in this sensitive area. Health institutions should also prioritize ongoing professional development, reflective practice, and emotional support mechanisms – such as supervision and debriefing sessions – to help nurses manage the emotional demands associated with suicide prevention at work. At the policy level, suicide prevention should be embedded into national (nursing) strategies and clinical guidelines, recognizing the unique contributions nurses make across care settings.

From a research perspective, further studies are needed to evaluate the effectiveness of nursing-led suicide prevention interventions and to examine the contextual factors that facilitate or hinder their implementation. Additionally, participatory research involving nurses and individuals with lived experience of suicidality may help develop more person-centered, culturally appropriate, and ethically grounded approaches to care. Strengthening the evidence base in these areas is critical for advancing nursing practice and ensuring the sustainability and impact of suicide prevention efforts worldwide.

## Conclusion

Given the critical importance of suicide prevention for individuals experiencing suicidality, a desire to die, or requesting MAiD, it is essential to clearly delineate the roles and responsibilities of caregivers. This scoping review provides a comprehensive overview of the diverse and multifaceted roles nurses play in suicide prevention within adult care settings across international contexts. The analysis of *n* = 25 sources of evidence reveals that nursing responsibilities span six key roles: 3.3.1 *Early Detection and Risk Assessment*, 3.3.2 *Relationship-Building and Communication*, 3.3.3 *Education and Advocacy*, 3.3.4 *Risk Management*, 3.3.5 *Interdisciplinary Collaboration*, and 3.3.6 *Personal and Professional Self-Management*. These roles demonstrate the depth of nursing engagement at all levels of suicide prevention – universal, selective and indicated – and highlight the unique position of nurses to contribute meaningfully to the identification, intervention, and long-term support of individuals at risk. In light of the discussions surrounding assisted suicide in many countries across the world and its implementation, the topic of suicide prevention and the role and responsibility of nurses in all settings and care relationships is becoming increasingly relevant.

However, the findings also point to the need for greater structural support, targeted education, and clear protocols to strengthen nurses’ competence and responsibility in this complex area of care. Future research and policy development should prioritize the integration of suicide prevention competencies into nursing education and practice frameworks, as well as foster supportive environments that enable nurses to carry out these critical roles effectively and sustainably.

## Supplementary Information

Below is the link to the electronic supplementary material.

Supplementary Material 1

Supplementary Material 2

Supplementary Material 3

Supplementary Material 4

## Data Availability

Generated data during the review process – including the search strategy, selection and extraction guidance as well as a list of excluded sources of evidence – are provided in this article (see Supplementary Files 1–4).

## Footnotes

1 A preliminary search in MEDLINE and CINAHL was conducted in September 2024 to identify reviews on this topic.

## Abbreviations

APNA: American Psychiatric Nurses Association
GHVN: General Home Visiting Nurses ICN: International Council of Nurses
RNAO: Registered Nurses’ Association of Ontario
MAiD: Medical Assistance in Dying
WHO: World Health Organization
