# Supplementary material for: Nurses’ roles and responsibilities in suicide prevention: a scoping review
Source: BMC Nurs. 2025 Oct 22;24:1308. doi: 10.1186/s12912-025-04009-5 (PMC12542392; doi:10.1186/s12912-025-04009-5)
Supplement: Supplementary file 1 — Supplementary Material 1 [file 12912_2025_4009_MOESM1_ESM.pdf]

Search component	MEDLINE via EBSCOhost	CINAHL via EBSCOhost	PsycInfo via EBSCOhost
**Population/ Participants**	(TI nurs* OR AB nurs* OR MH Nurses OR MH Nursing)	(TI nurs* OR AB nurs* OR MH Nurses)	(TI nurs* OR AB nurs* OR DE Nurses OR DE Nursing)
	**AND**	**AND**	**AND**
**Concept**	(TI suicid* OR AB suicid* OR TI "desire to die" OR AB "desire to die" OR TI "wish to die" OR AB "wish to die" OR TI "wish to hasten death" OR AB "wish to hasten death" OR TI "desire for hastened death" OR AB "desire for hastened death" OR TI "medical assistance in dying" OR AB "medical assistance in dying" OR TI "MAiD" OR AB "MAiD" OR TI "medical aid in dying" OR AB "medical aid in dying" OR TI "assisted suicide" OR AB "assisted suicide" OR TI "physician-assisted suicide" OR AB "physician-assisted suicide" OR TI "assisted dying" OR AB "assisted dying" OR TI euthanasia OR AB euthanasia)<br><br>AND<br><br>(TI prevent* OR AB prevent* OR TI manag* OR AB manag* OR TI "suicide prevention" OR AB "suicide prevention" OR MH Suicide Prevention)<br><br>AND<br><br>(TI role# OR AB role# OR MH Nursing Role OR TI duty OR AB duty OR TI duties OR AB duties OR TI obligation# OR AB obligation# OR TI responsibilit* OR AB responsibilit* OR TI engag* OR AB engag* OR TI collaborat* OR AB collaborat* OR TI relation* OR AB relation* OR MH Nurse-Patient Relations OR TI interaction# OR AB interaction# OR TI connection# OR AB connection# OR TI "working alliance" OR AB "working alliance" OR TI contact# OR AB contact#)	(TI suicid* OR AB suicid* OR TI "desire to die" OR AB "desire to die" OR TI "wish to die" OR AB "wish to die" OR TI "wish to hasten death" OR AB "wish to hasten death" OR TI "desire for hastened death" OR AB "desire for hastened death" OR TI "medical assistance in dying" OR AB "medical assistance in dying" OR TI "MAiD" OR AB "MAiD" OR TI "medical aid in dying" OR AB "medical aid in dying" OR TI "assisted suicide" OR AB "assisted suicide" OR TI "physician-assisted suicide" OR AB "physician-assisted suicide" OR TI "assisted dying" OR AB "assisted dying" OR TI euthanasia OR AB euthanasia)<br><br>AND<br><br>(TI prevent* OR AB prevent* OR TI manag* OR AB manag* OR TI "suicide prevention" OR AB "suicide prevention" OR MH Suicide Prevention)<br><br>AND<br><br>(TI role# OR AB role# OR MH Nursing Role OR TI duty OR AB duty OR TI duties OR AB duties OR TI obligation# OR AB obligation# OR TI responsibilit* OR AB responsibilit* OR TI engag* OR AB engag* OR TI collaborat* OR AB collaborat* OR TI relation* OR AB relation* OR MH Nurse-Patient Relations OR TI interaction# OR AB interaction# OR TI connection# OR AB connection# OR TI "working alliance" OR AB "working alliance" OR TI contact# OR AB contact#)	(TI suicid* OR AB suicid* OR TI "desire to die" OR AB "desire to die" OR TI "wish to die" OR AB "wish to die" OR TI "wish to hasten death" OR AB "wish to hasten death" OR TI "desire for hastened death" OR AB "desire for hastened death" OR TI "medical assistance in dying" OR AB "medical assistance in dying" OR TI "MAiD" OR AB "MAiD" OR TI "medical aid in dying" OR AB "medical aid in dying" OR TI "assisted suicide" OR AB "assisted suicide" OR TI "physician-assisted suicide" OR AB "physician-assisted suicide" OR TI "assisted dying" OR AB "assisted dying" OR TI euthanasia OR AB euthanasia)<br><br>AND<br><br>(TI prevent* OR AB prevent* OR TI manag* OR AB manag* OR TI "suicide prevention" OR AB "suicide prevention" OR DE Suicide Prevention)<br><br>AND<br><br>(TI role# OR AB role# OR DE Professional Role OR TI duty OR AB duty OR TI duties OR AB duties OR TI obligation# OR AB obligation# OR TI responsibilit* OR AB responsibilit* OR TI engag* OR AB engag* OR TI collaborat* OR AB collaborat* OR TI relation* OR AB relation* OR DE Interpersonal Relationships OR DE Therapeutic Processes OR TI interaction# OR AB interaction# OR TI connection# OR AB connection# OR TI "working alliance" OR AB "working alliance" OR TI contact# OR AB contact#)
**Search results**	564	524	375
