# Supplementary material for: Nurses’ roles and responsibilities in suicide prevention: a scoping review
Source: BMC Nurs. 2025 Oct 22;24:1308. doi: 10.1186/s12912-025-04009-5 (PMC12542392; doi:10.1186/s12912-025-04009-5)
Supplement: Supplementary file 2 — Supplementary Material 2 [file 12912_2025_4009_MOESM2_ESM.pdf]

Screening guidance

Objective	Aim:	Research question:
	To identify nurses' roles and responsibilities in suicide prevention across adult care settings within an international context.	What are nurses' roles and responsibilities in suicide prevention across adult care settings within an international context?
**Criteria**	**Inclusion**	**Exclusion**
Population/ Participants	Perspectives of nurses with varying levels of professional qualification or nurses were actively involved as participants	Other healthcare professionals alongside nurses and the data specific to nurses could not be extracted and analyzed separately
Concept	Focus on roles and responsibilities of nurses in suicide prevention, including the specific context of requests for Medical Assistance in Dying (MaiD), assisted suicide, or euthanasia	suicidality among nurses themselves<br>Focus on training or educational needs of nurses related to specific interventions or programs<br>Focus on the testing or evaluation of particular interventions, programs, or instruments
Context	Focus on adult care settings in an international context	Non-care settings<br>Focus exclusively on suicide prevention among children or adolescents
Types of evidence sources	empirical studies – including qualitative, quantitative, mixed-method research, and case reports – published in peer-reviewed journals<br>reviews incorporating peer-reviewed empirical studies<br>guidelines, standards, policy papers, discussion papers, and professional codes	-
Language	English and German	-
Publication date	published in any year	-
