# Supplementary material for: Nurses’ roles and responsibilities in suicide prevention: a scoping review
Source: BMC Nurs. 2025 Oct 22;24:1308. doi: 10.1186/s12912-025-04009-5 (PMC12542392; doi:10.1186/s12912-025-04009-5)
Supplement: Supplementary file 3 — Supplementary Material 3 [file 12912_2025_4009_MOESM3_ESM.docx]

| **Reference** | | **Included after Full-Text Screening** | **Included in Data Extraction and Analysis** |
| --- | --- | --- | --- |
| **No.** | **Author(s), Year, Title** |  |  |
| [1] | Betz et al., 2019. Counseling suicidal patients about access to lethal means: attitudes of emergency nurse leaders. | yes  no  Reason for Exclusion:  wrong concept | yes  no |
| [2] | Clua-García et al., 2021. Suicide care from the nursing perspective: a meta-synthesis of  qualitative studies | yes  no | yes  no  Reason for Exclusion:  Review |
| [3] | Collins & Cutcliffe, 2003. Addressing hopelessness in people with suicidal ideation: building upon the therapeutic relationship utilizing a cognitive behavioural approach. | yes  no  Reason for Exclusion:  wrong concept | yes  no |
| [4] | Cutcliffe et al., 2007. Reconnecting the person with humanity: how psychiatric nurses work with suicidal people. | yes  no  Reason for Exclusion:  wrong population | yes  no |
| [5] | Duffy et al., 2003. Focus. Exploring suicide risk and the therapeutic relationship: a case study approach. | yes  no  Reason for Exclusion:  wrong concept | yes  no |
| [6] | Garand et al., 2006. Suicide in older adults: nursing assessment of suicide risk. | yes  no | yes  no  Reason for Exclusion:  Review |
| [7] | Karlsson et al., 2021. Capturing the unsaid: nurses' experiences of identifying mental ill-health in older men in primary care – A qualitative study of narratives. | yes  no  Reason for Exclusion:  wrong concept | yes  no Reason: |
| [8] | Repper, 1999. A review of the literature on the prevention of suicide through interventions in accident and emergency departments. | yes  no Reason: | yes  no  Reason for Exclusion:  Review |
| [9] | Poreddi et al., 2021. Attitudes of under graduate nursing students to suicide and their role in caring of persons with suicidal behaviors. | yes  no  Reason for Exclusion:  wrong concept | yes  no Reason: |
| [10] | Shin et al., 2021. A poststructural analysis: Current practices for suicide prevention by nurses in the emergency department and areas of improvement. | yes  no | yes  no  Reason for Exclusion:  Review |
| [11] | Siau et al., 2019. Malaysian nurses' attitudes toward suicide and suicidal patients: a multisite study. | yes  no  Reason for Exclusion:  wrong concept | yes  no |
| [12] | Sun et al., 2005. Suicide: a literature review and its implications for nursing practice in Taiwan. | yes  no | yes  no  Reason for Exclusion:  Review |
| [13] | Sun et al., 2006. Patients and nurses' perceptions of ward environmental factors. and support systems in the care of suicidal patients. | yes  no  Reason for Exclusion:  wrong population | yes  no |
| [14] | Talseth & Gilje, 2011. Nurses’ responses to suicide and suicidal patients: a critical interpretive synthesis. | yes  no | yes  no  Reason for Exclusion:  Review |
| [15] | Troya et al., 2024. Healthcare practitioners' views of self‐harm management practices in older adults in Ireland: a qualitative study. | yes  no  Reason for Exclusion:  wrong population | yes  no |
| [16] | Vedana et al., 2018. The meaning of suicidal behaviour from the perspective of senior nursing undergraduate students. | yes  no  Reason for Exclusion:  wrong concept | yes  no |

**References**

1. Betz ME, Brooks-Russell A, Brandspigel S, Novins DK, Tung GJ, Runyan C. Counseling suicidal patients about access to lethal means: attitudes of emergency nurse leaders. J Emerg Nurs. 2018;44(5):499–504. doi:10.1016/j.jen.2018.03.012.
2. Clua-García R, Casanova-Garrigós G, Moreno-Poyato AR. Suicide care from the nursing perspective: a meta-synthesis of qualitative studies. J Adv Nurs. 2021;77(7):2995–3007. doi:10.1111/jan.14789.
3. Collins S, Cutcliffe JR. Addressing hopelessness in people with suicidal ideation: building upon the therapeutic relationship utilizing a cognitive behavioural approach. J Psychiatr Ment Health Nurs. 2003;10(2):175–185. doi:10.1046/j.1365-2850.2003.00573.x.
4. Cutcliffe JR, Stevenson C, Jackson S, Smith P. Reconnecting the person with humanity: how psychiatric nurses work with suicidal people. Crisis. 2007;28(4):207–210. doi:10.1027/0227-5910.28.4.207.
5. Duffy D. Exploring suicide risk and the therapeutic relationship: a case study approach. NT Research. 2003;8(3):185–199. doi:10.1177/136140960300800304.
6. Garand L, Mitchell AM, Dietrick A, Hijjawi SP, Pan D. Suicide in older adults: nursing assessment of suicide risk. *Issues Ment Health Nurs*. 2006;27(4):355–370. doi:10.1080/01612840600569633.
7. Karlsson J, Hammar LM, Kerstis B. Capturing the unsaid: nurses' experiences of identifying mental ill-health in older men in primary care – A qualitative study of narratives. Nurs Rep. 2021;11(1):152–163. doi:10.3390/nursrep11010015.
8. Repper J. A review of the literature on the prevention of suicide through interventions in accident and emergency departments. J Clin Nurs. 1999;8(1):3–12. doi:10.1046/j.1365-2702.1999.00218.x.
9. Poreddi V, Anjanappa S, Reddy S. Attitudes of under graduate nursing students to suicide and their role in caring of persons with suicidal behaviors. Arch Psychiatr Nurs. 2021;35(6):583–586. doi:10.1016/j.apnu.2021.08.005.
10. Shin HD, Price S, Aston M. A poststructural analysis: current practices for suicide prevention by nurses in the emergency department and areas of improvement. J Clin Nurs. 2021;30(1-2):287–297. doi:10.1111/jocn.15502.
11. Siau CS, Wee LH, Adnan TH, Yeoh SH, Perialathan K, Wahab S. Malaysian nurses' attitudes toward suicide and suicidal patients: a multisite study. J Nurses Prof Dev. 2019;35(2):98–103. doi:10.1097/NND.0000000000000520.
12. Sun FK, Long A, Boore J, Tsao LI. Suicide: a literature review and its implications for nursing practice in Taiwan. J Psychiatr Ment Health Nurs. 2005;12(4):447–455. doi:10.1111/j.1365-2850.2005.00863.x.
13. Sun FK, Long A, Boore J, Tsao LI. Patients and nurses' perceptions of ward environmental factors and support systems in the care of suicidal patients. J Clin Nurs. 2006;15(1):83–92. doi:10.1111/j.1365-2702.2005.01232.x.
14. Talseth AG, Gilje FL. Nurses' responses to suicide and suicidal patients: a critical interpretive synthesis. J Clin Nurs. 2011;20(11-12):1651–1667. doi:10.1111/j.1365-2702.2010.03490.x.
15. Troya MI, Lonergan C, Cassidy E, Griffin E, Lovejoy SA, Mughal F, et al. Healthcare practitioners' views of self-harm management practices in older adults in Ireland: a qualitative study. Int J Geriatr Psychiatry. 2024;39(7):e6116. doi:10.1002/gps.6116.
16. Vedana KGG, Pereira CCM, Dos Santos JC, Ventura C, Moraes SM, Miasso AI, et al. The meaning of suicidal behaviour from the perspective of senior nursing undergraduate students. Int J Ment Health Nurs. 2018;27(3):1149–1161. doi:10.1111/inm.12431.
