# Supplementary material for: Nurses’ roles and responsibilities in suicide prevention: a scoping review
Source: BMC Nurs. 2025 Oct 22;24:1308. doi: 10.1186/s12912-025-04009-5 (PMC12542392; doi:10.1186/s12912-025-04009-5)
Supplement: Supplementary file 4 — Supplementary Material 4 [file 12912_2025_4009_MOESM4_ESM.pdf]

Data Extraction Guidance

Entities to Extract	Description	Example
Reference	List the authors and the year the evidence source was published.	[Author/s] et al. [Year]
Country	Specify the country where the evidence source was published.	England
Type of evidence source	Indicate the type of evidence source. If applicable, state the study design or type of guideline/standard.	Qualitative Study
Population/Participants	Specify the population/participants that are included in the evidence source.	N = X Emergency Department Nurses
Context	Indicate the adult care setting on which the evidence source focuses. If applicable, identify a specific ward or unit.	Hospital, Psychiatric Ward
Concept	Summarize key findings related to the review question.	- ...<br>- ...
